# Bringing ethics into governance: the case of the UK COVID-19 contact tracing app

**DOI:** 10.1108/ijhg-04-2021-0042

**Published:** 2021-12-27

**Authors:** Gabrielle Samuel, Federica Lucivero

**Affiliations:** Department of Global Health and Social Medicine, King’s College London, London, UK; Nuffield Department of Population Health, Ethox and Wellcome Centre for Ethics and Humanities, University of Oxford, Old Road Campus, Oxford, UK

**Keywords:** Contact tracing app, Digital health, Ethics, NHSX, RRI, Governance, Pandemic, Preparedness

## Abstract

**Purpose:**

In April 2020, it was announced that NHSX, a unit of the UK National Health Service (NHS) responsible for digital innovation, was developing a contact tracing app that would offer a digital solution to managing the COVID-19 pandemic. Despite the urgency with which the app was developed, a clear commitment was made to designing the technology in a way that enshrined key ethical principles, and an ethics advisory board (EAB) was established to provide timely advice, guidance and recommendations on associated ethical issues. Alongside this, there were extensive criticisms of how NHSX adhered to ethical principles in the handling of the app development-criticisms that require empirical exploration. This paper explores how ethics was incorporated into decision-making during governance processes associated with the development of app.

**Design/methodology/approach:**

Interviews were conducted with those involved in the app’s development/governance, those with a consulting role associated with the app, or those who sat on the EAB.

**Findings:**

The EAB fulfilled an important role by introducing ethical considerations to app developers. Though at times, it was difficult to accommodate key ethics principles into governance processes, which sometimes suffered from little accountability.

**Originality/value:**

While several articles have provided overviews of ethical issues, or explored public perceptions towards contact tracing apps, to the best the authors, knowledge this is the first empirical piece analysing ethics governance issues via stakeholder interviews.

## Introduction

On 12 April 2020, Matt Hancock, UK Secretary of State for Health and Social Care, announced that NHSX, a unit of the UK National Health Service (NHS) responsible for digital innovation, was developing a contact tracing app that would offer a digital solution to managing the COVID-19 pandemic in England and Wales. The app, similar to apps that had or would soon be developed by other countries, would work by tracing individuals who had come into contact with those who had reported symptoms or tested positive for the virus and request that they self-isolate. The app was trailed on the Isle of Wight in May/June 2020.

Despite the urgency and speed with which the app was developed, a clear commitment was made to designing the technology in a way that enshrined key ethical principles. An ethics advisory board (EAB) was established that was described by the CEO of NHSX and the senior responsible owner of the app project as “crucial” to providing timely advice, guidance and recommendations on ethical issues associated with the app’s development (Gould and Lewis, 2020).

NHSX’s commitment to ethics reflects the contemporary landscape of both science and health governance that aims to pay increasing attention to ethical issues at the policy level. This attention to ethics in public policy has been shown to have a vital role in creating a space for moral thinking during decision-making (Hine, 2010), and various expert bodies, especially ethics advisory bodies, have become institutionalised at the national level (Pastovrh and Mali, 2015). However, as organisations specifically move to incorporate ethics into governance structures, sociologists have become concerned that some organisations or institutions presenting themselves as “ethical” entities do not always reflect a space within which moral thinking occurs (Fleming and McNamee, 2005). This paper contributes to this sociological literature, which has emerged in different fields. Historically it has been particularly prominent in the health governance arena – particularly in biobanking. In this field, the “ethical work” conducted (and displayed) by organisations has been argued to be more strategic, and more related to the organisation’s politics (Petersen, 2005), with the “ethical presence” primarily being about fostering public confidence and legitimisation without a genuine engagement of the organisation with ethical principles (Jasanoff, 2005). In fact, Hoeyer and Tutton (2005) argue that the term “ethics” gained a specific institutionalised purpose in biobanking by “*demonstrating that ethical problems are attended to*” (p. 386). For these and other scholars, concerns revolved around the way in which organisations focused on prominent ethical concepts such as consent, at the expense of other less attended issues. More recently, a growing instrumentalisation of ethical language has been highlighted with relation to technology companies in the big data and artificial intelligence arena. This has recently become known as “ethics washing” (Wagner, 2018). Here, scholars are concerned that such companies aim to appear to be ethical entities to avoid self-regulation and accountability (Wagner, 2018). Examples of ethics washing, argue scholars, include focussing on various ethical issues at the expense of deeper questioning around the broader impacts of systems on society (Bietti, 2020). Other scholars have argued that some institutions can in fact do both–describe themselves as ethical, as well as act ethically, though acting ethically here will always be within the confines of the political and/or economic premise of the institution. This means that ethical deliberation will be restricted by institutional requirements, rather than related to broader questions around social value (Samuel and Farsides, 2018; Datta Burton *et* *al*., 2021).

This paper contributes to this literature by analysing NHSX’s commitment to ethics. Specifically, it aims to explore whether, similar to the tensions described in the literature, this commitment created a space for moral thinking to align decision-making processes with ethical principles and societal goals; or whether it was more related to the instrumentalisation of ethics.

In fact, the NHSX was questioned for a lack of transparency in the way ethical advice was sought and implemented in the decision-making process around the app, as well as with relation to a lack of clarity about the nature of private partnerships between NHSX and commercial companies, and the time and purpose limitations associated with the use of the app (AdaLovelace Institute, 2020; House of Commons House of Lords Joint Committee on Human Rights, 2020; Kerr, 2020). These concerns were also raised by other scholars at the international level, with calls being made for apps to be developed using principles of transparency, openness, public engagement, trustworthy governance, accountability, equity and non-discrimination (Kahn and Johns Hopkins Project on Ethics and Governance of Digital Contact Tracing Technologies, 2020; Lucivero *et* *al*., 2020; Parker *et* *al*., 2020).

Specifically, the aim of this paper is to empirically explore the processes established to ensure enshrined ethical principles were embedded into the app’s development and governance, or in other words, how ethics was incorporated into decision-making during governance processes. Publicly available information about the working of the EAB is available in its terms of reference and final report (Montgomery, 2020; NHSX, 2020). To better understand how ethics was incorporated into the development process, we conducted eight interviews with professional stakeholders who had a role associated with the app’s development. Our research question was: what were interviewees’ views and experiences of how ethical reflection was incorporated into governance processes and decision-making during the generation of the NHSX app? Our findings show how the EAC fulfilled an important role by introducing ethical considerations to app developers. However, they also show how they sometimes found it difficult to have influence in accommodating key principles of ethics into governance processes, which suffered from little accountability.

## Methods

Eight interviews were conducted in June–August 2020 with those involved with the development/governance of the app, those who had a consulting role associated with the app, or those who sat on the EAB. Interviewees were recruited via identification of email addresses online and by snowballing. Identifying potential interviewees was complicated by the lack of online contact information about those involved in the app development and/or governance process. Furthermore, the politically sensitive nature of app development at the time meant that some individuals had concerns about speaking to us. In total, 26 individuals were asked to participate, giving a response rate of approximately 32%. While we only interviewed 8 individuals, and this is a limitation of the study, interviews were extremely rich and lasted between 57–90 min. Interviews were digitally-recorded and transcribed. They explored interviewees’ practices, views, beliefs and experiences associated with the development and governance of the app (see appendix). Analysis of the interviews was inductive and interactive (Strauss, 1987) and involved authors reading the transcripts independently, inductive coding by GS and discussing findings at two virtual meetings to generate themes. Given the political context of the app development, and the need to maintain strict confidentiality, no further information can be provided about our interviewees’ status. Furthermore, small changes have been made to some extracts to hide interviewees’ identities. At times, this may have led to extracts losing some detail, and seeming more abstract than would usually be presented, though care was taken to ensure changes did not detract from meanings generated.

Ethics approval: King’s College London research ethics office: MRA-19/20–19,251.

## Findings

### A sense of urgency to save lives

One of the most striking, yet unsurprising aspects of interviewees’ discussions, was the sense of urgency within which those involved with the development and/or governance of the NHSX app described the initiative. While some interviewees reported working through other national emergencies, this particular emergency was described as especially difficult and distinctive because of its scale (interviewee 6). Interviewees spoke at length about the speed with which the app was developed, the amount of work that was conducted over a short space of time and the “huge amount of pressure” (interviewee 5) individuals were under. This seeming urgency to develop an app at speed was based around the belief that it would bring benefit by helping to contain the virus and the worry that traditional manual contact tracing would not be fast enough to control the epidemic. In fact, in a public announcement at the launch of the NHSX app, the technology had been described as having “the potential to save lives” (Gould and Lewis, 2020), and this was a phrase that was used by a number of interviewees during the interviews as a justification for some of the eventual shortcomings of the app. Interviewee 4, for example, spoke about how the rush to develop the app meant that technical corners were cut, making the technology less flexible to accommodating future policy changes than perhaps would have otherwise been the case, but that this was justified on the basis of “saving the country”;

we were in a mad rush to try and get this thing launched on the Isle of Wight so we charged ahead and did the app… .[…]…if we were not in so much of a rush we would have invested more time in being able to be more flexible later on.… we were focused on just getting one or two versions of the app out in order to *save* the country, rather than investing in the process of being able to launch new versions of the app almost on a nightly basis.

Interviewee 8 described how this sense of urgency to save lives raised broad concerns about the context of the app’s development. In the extract below this interviewee explains how the app was being developed before the wider contact test and trace programme had been established. This led to a situation in which the app had become removed from its context of use;

at the time we started building this … the wider test and trace programme had not been set up. So it was a disembodied technical thing that was waiting to dock into something.

Perhaps it was this missing context of manual tracing -perceived by this interviewee-that so much emphasis was placed on this technology as a “panacea” to control the spread of the virus (interviewee 8). However, this approach, of separating the technology from its context of use soon became a hindrance for those working so tirelessly on the app; “*it would have been better*”, explained interviewee 3, “*to have a considered test and trace as the core issue, of which the app is the little bauble hanging off it*”. It was not until later, that the political discourse around the app changed, the development of the app was subsumed under the governance of the NHS test and trace programme, and the app was promoted as being the “cherry on the cake”;

the governance strategy only arrived at some point in time … And then it [the app] was fitted into this larger track and trace structure. Then essentially towards the end of that there was a change in teams whereby all of those people who’d essentially built the initial app and responded to the emergency were sort of pulled to the side, and Dido Harding and other track and trace people took over … (interviewee 6).

While on the one hand, the focus on the app as a means to save lives was problematic because it removed the app from its context of use, on the other hand, the late integration of the app in the broader programme alienated those who worked relentlessly for the success of the app, leaving them feeling disenfranchised.

### The ambiguous role of the ethics advisory board

The EAB was established soon after the app initiative commenced, with the first meeting taking place on 2nd April 2020. The EAB’s purpose was to ensure that the development of the app helped control the pandemic and return people to normal life more rapidly whilst operating in line with ethical requirements and being transparent and open to public scrutiny; its terms of reference were made openly available (NHSX, 2020). A report on the board’s functioning has also since been published (Montgomery, 2020).

Interviewees explained how the EAB sat somewhere between their perceptions of other ethics boards – i.e. between, on the one side, a committee with gatekeeper function, such as a research ethics committee, or on the other side, an independent ethics board outside of an organisation, which described and reviewed ethics issues. Interviewee 3 felt that because of the rush with which it was set up, the EAB’s role was never made entirely unclear. This interviewee remarked;

often you are there [on boards] partly to say to them have you thought of this, I can put you in touch with this person …. So you’re there for your expertise and that works well .… That’s completely different than being the equivalent of in a university medical ethical trial board … where you’ve actually got a ‘stop you can’t do this’ power, that’s effective. But this really fell in the middle … I do’t think its role was ever clear.

For a number of interviewees, this middle position had clear advantages: whilst it might not be governing nor independent, it was perceived to be embedded into the governance hierarchy such that the EAB’s Chair was described as “*regularly speaking*” to those involved in the app’s governance, “*creating a dialogue*” to ensure “*ethics is on their radar*” (interviewee 1). This, in turn, was perceived to allow EAB discussions to be fed into the app’s steering committee. Interviewee 1 provided different examples of the influential and substantial role they perceived the Board to have had in decision-making processes;

the self-reporting capability of the app [a person who develops symptoms can trigger an app alert], really was not being seen as problematic by the app team until we raised it. But once we raised it, they took that … seriously;

we weren’t the only people pushing that [the app should be considered as part of the wider test and trace programme], and we weren’t necessarily the major pressure, but that’s a response to something that we were raising.

Interviewee 4 similarly spoke about the crucial importance of the EAB’s role. For this interviewee, the presence of the committee provided a “sounding board” for the consideration of ethical issues. This was particularly important for those making decisions about the app’s development because the multitude of other conflicting interests (engineering, technical, public health, political) that was at play;

whereas they [app developers] were being pulled by all sorts of people to bring in geolocation data, age, ethnicity, gender, co-morbidities[…] what the Ethics Advisory group helped them do really, was to adjust the centre of gravity about what would be acceptable … The EAB were a useful sounding board for ideas throughout the process.

Interviewee 8 explained that, in this way, the EAB functioned to put ethics “at the back of the mind” of people who developed the app.

However, for some interviewees, this middle-of-the-road function of the EAB was more an indicator of the speed with which the committee was established, which gave little consideration to its purpose. This intermediate role frustrated these interviewees who were concerned about the Board’s lack of decision-making power, meaning that ethics could only be given as advice rather than having to be listened to; “*the only power the advisory board members had was really to quit as far as I see it. They could give all the best advice they wanted and that may or may not have been listened to*” (interviewee 7). This was not trivial, on a couple of occasions, some EAB members threatened to resign. Moreover, the lack of EAB day-to-day involvement in the development of the app was also viewed by some as problematic: “*if they could*’*ve been more involved on a day-to-day basis it would*’*ve been beneficial*” (interviewee 8). For example, the EAB final report highlights how the EAB was never provided with the level of detail they asked for concerning the technical data from the tests and trial as they would expect as an internal board who can review confidential material (Montgomery, 2020, p. 14).

Beyond concerns with the way the EAC functioned, interviewees spoke about the lack of accountability of governance structures and lack of transparency in communication strategies. We discuss each.

### Lack of accountability of governance structures

Political practices were perceived by nearly all interviewees to be at the heart of much decision-making associated with the app. The EAB produced three formal documents of advice during its standing, and although all three pieces included the need for accountability issues to be addressed (Montgomery, 2020, appendices 6, 8, 9), interviewees provided a range of specific examples in which politics had taken, or had *seemingly* taken centre-stage in these decision-making processes. Through these examples, a lack of governance infrastructure around the app, or at least the lack of *enforcement* or *accountability* of governance structures was exposed. For example, some interviewees pointed to their perception that decisions about the app were entangled within the tensions between the NHSX, Public Health England, the government and the wider contact tracing initiative more generally. Other interviewees indicated their concerns about how decisions relating to the app were sometimes unaccountable; “*you have a feeling that a lot of these organisations are mates essentially. And I think that*’*s actually genuinely been one of the problems with the government*’*s handling of this scheme* … *it was all very cosy* (interviewee 3).

Even when governance structures were in place, interviewees worried about how “*people [still] just behaved badly*”. For instance, although the EAB provided formal advice about what ethical aspects should be considered during any decision regarding a centralised or decentralised approach to the app’s data collection (Montgomery, 2020, pp. 42–43), some interviews suggested this advice was not always adhered to. Interviewee 2 was particularly anxious about how the processes developed for evaluating an arbitrated field test between the NHSX and the Google/Apple app were not followed in line with the governance structures put in place, and that it was this decision-making that partly led to the eventual retraction of the NHSX app;

the data came back from one of the two companies [each testing one of the apps], I do not know which, but it went straight to Baroness Dido Harding … straight to Matt Hancock, and they stood there in front of the box [TV cameras] before that plan was followed and they delivered th[e] statistic [that led to halting the Isle of Wight trial]. And that is still a statistic that the engineers dispute ….

Interviewee 4 similarly described a “murky” governance around the decision to switch from the NHSX-developed app to one that used the Google/apple API. This interviewee was also frustrated that ordinary channels of governance that oversaw decision-making were not always followed;

the governance of [the decision to switch to Google] -it’s very murky, it was some kind of Saturday afternoon thing. ….Normally for such a momentous decision you’d have a, what’s called a submission, which lists all the pros and the cons and the risks involved, and we’d get legal opinion and IT opinion, none of that happened.

The perceived lack of appropriate governance structure was often explained and justified in relation to the perceived urgency of the situation. Interviewee 4 explained that it was sometimes difficult to “*adhere to the normal principles of good sound governance*” [because] “*we did not have time to do them because things were just being done in such a mad rush*”. As other interviewees explained, because of the “*urgency of the situation*” oversight was not always at the forefront of people’s minds as much as if, for example, a digital technology had been developed for a particular disease in ordinary circumstances (interviewee 5).

The urgency of the pandemic almost seemingly gave ethical permission for unaccountable decisions to be made despite calls by the EAB to include the need for accountability in decision-making (Montgomery, 2020). In fact, some of the decisions being made about the app were often excused by interviewees as a consequence of the rushed and urgent context of the initiative, whose focus was very much on saving lives. Speaking about a specific decision, interviewee 8 remarked “*I do not think this was an intentional thing, I think this was a function of how quickly the original team was set up*”. Similarly, there was an acceptance that, because of the speed with which the governance infrastructure had been established it was understandable that when choosing who should be placed on the app’s development team, individuals could rely on personal contacts and technical networks; “*I suggested [confidential]* … *and when you are in a hurry you need to use your technical network to get the best people that were there*” (interviewee 6).

### Lack of transparency in communication strategies

Despite the EAB’s formal advice calling for openness and transparency (Montgomery, 2020), most interviewees spoke about being frustrated with a perceived lack of openness with which the government conducted itself; “*you did get a perception as things began to get slightly less emergency-ish, that the government was essentially just ultra-cautious about revealing anything*” (interviewee 3). For these interviewees, this was related to a hierarchy of power (“*they sort of forced people into this hierarchy*” (interviewee 2)). Interviewee 2 reflected on being misled about some information on a technological issue with the app – an issue that has also been mentioned in the EAB published report (Montgomery, 2020). Briefly, this interviewee had been informed this issue was fixed, but from their understanding it had not, rather it had been buried, and later somebody had “coughed up” to admit the issue was still there;

there was a plan, that it was flagged that there was still an engineering problem with the smart phone problem, right. This is after we’d been told by the developers that they’d found a work around, then somebody coughed up and said ‘oh actually, maybe there is still a problem here’.[…] …I can tell you that what was said about the technological problems was misleading (identification withheld).

Given the “low trust”, political environment within which they perceived they were working (interviewee 6) and resonating with the advice given by the EAB (Montgomery, 2020, p. 42), some interviewees were particularly concerned about the effect of the government’s choice to remain “silent” about the app in public discourse. For interviewee 8, this “awful” communication meant that “everything became rumour and conjecture”, leading to a situation in which there was no control over the public narrative;

Instead of getting on the front foot and saying here is what we are building … here is how it’s going to work, you’ve 3 months of rumour and conjecture …. we had not controlled the narrative.

This rumoured narrative focused on privacy concerns. Interviewee 6 explained how this scenario should have been better prepared for and indeed prioritised;

we should have anticipated the privacy debate and been better set up and prepared for it… […]… it would have made sense to have devoted effort at the cost of something else.

In fact – and resonating with the way the governance infrastructure of the app had been established – interviewee 4 spoke about the “comms” being more of an after-thought than something that was considered at the start of the app’s development process. In fact, interviewee 5 perceived a general broader need for ethical preparedness of oversight mechanisms for pandemic emergency situations was required;

When we get into an emergency … we tend to not learn from previous experience .… I think we need to focus … more in preparedness, and thinking about that, between emergencies … to enable us to respond in an ethical and fair, but also effective way next time this happens.

Transparency however was not considered key by everyone. One interviewee pointed out that transparency should come after more important values, such as “saving people’s lives” (interviewee 6).

## Discussion

Our findings contribute to the sociological literature that has empirically explored different organisations’ commitments to act as ethical entities – especially organisations in the health arena, as well as more recently in the technology arena. Similar to this literature, our findings illustrate that the NHSX’s commitment to ethics when designing and developing the contact tracing app did not *necessarily* lead to a space in which institutional ethical decision-making occurred (Hoeyer and Tutton, 2005; Petersen, 2005; Wagner, 2018). Specifically, our findings highlight that, on the one hand, those who contributed to the app’s development worked furiously and tirelessly as they hoped to develop a technology to help manage the pandemic. For these individuals, their ultimate goal was to save lives, and they were motivated by the vision of achieving this. Tied to their practices, was a consideration of ethics – legitimised through the EAB. This board fulfilled an important role by placing ethical considerations “*at the back of people*’*s minds*” of those making decisions about the development, acting as what was described as a sounding board. In this way, the EAB had a role to play in ensuring ethics was a consideration for those involved in the governance of the app and had some influence on decision-making. However, on the other hand, there was a sense that even though the EAB had a published terms of reference (NHSX, 2020), at times its members felt the board’s role was poorly defined-a point made elsewhere about other EABs more generally (Mali *et* *al*., 2012). This meant it was difficult to accommodate key ethics principles into governance processes, which sometimes suffered from little accountability. We note three reflections.

First, the EAB accomplished a laudable achievement by bringing ethical issues to the forefront for those developing and governing the app and bringing about some changes to practice. It does, however, call into question whether this micro level ethics role imparted by the EAB (bringing ethics to the forefront of people’s minds) is sufficient when considering ethics governance in general i.e. considering macro level organisational ethics issues related to accountability and governance processes. This is because organisational ethics is distinguished from the morals of those working within an organisation (Wieland, 2001)- in fact there is a distinction between the morals of an individual person (individual ethics), the morals of an individual within a given role (management ethics) and the morals of an organisation (governance ethics) (Wieland, 2001). This was further complicated in the case of the app because it was not clear what the relevant organisation was- NHSX was the host, but the decision-making “organisation” was the government. While the EAB seemed to promote individual reflexivity in terms of people’s own norms and beliefs about the development of the app, there was less evidence (though still some) that the EAB had a similar influence on the macro-organisational ethics exemplified by governance processes, such as accountability, openness and transparency. Indeed, although the EAB set out the need for such principles to be incorporated into governance processes, our interviewees sensed that governance mechanisms often fell short in terms of including them. As pointed out in the literature such attributes should be key not only for ethics committees (Hermerén, 2009), but also to ensure ethical governance more generally i.e. the set of processes, procedures and values designed to ensure the highest standards of organisational behaviour beyond simply good (i.e. effective) governance (Winfield, 2018). Looking forward, integrating ethics advisory committees into the organisational governance structure, such that representatives have an integral role (responsibility) in organisational decision-making, is one way to help ensure appropriate ethical governance [1].

In fact, more generally, to ensure ethical governance, and to move towards the inclusion and operationalisation of ethics at the macro level, would require mechanisms to be created to move ethics from “the back of people’s mind” to specific requirements in the app development and decision-making process. Such operationalisation of ethical reflection calls for caution against seeing ethics as a source of *solution* to complex, uncertain and morally problematic situations. In fact, the tools used in the ethical reflection are philosophical tools rarely effective to provide quick solution, but more often helpful to diagnose problems, disentangle different positions and show internal contradictions. One way to operationalise ethics without falling into a solutionist pitfall is to include its disentangling tools at different stages of innovation: to define problems at the beginning of the process; to explore possibilities, moral issues and implications of decisions during the decision-making process; and to assess the final product and explore ways to mitigate emerging problems in the evaluation phase. Therefore, while there is a need to operationalise ethics as part of the governance structure, it is also important to be realistic about what we can and should ask ethics to do; ethics can only support ethical thinking, guidance must not be rigid, but allow flexibility (Montgomery and Williams, 2019). This is an important point, given that many ethics bodies are often established to provide a solution to complex problems that emerge through science and technology developments. Ethics should be viewed as a process of governance – a way of doing and thinking about issues, rather than a solution.

Second, and connected to above, while there was a sense that ethics was seemingly considered at all stages of app development, it was not tightly weaved into the day-to-day processes. While ethical principles were stressed by the EAB (communication, accountability, openness, etc.), and likely well established and recognised by actors (even if they were not always enacted), ethics is more than just knowing and applying principles, it’s more about day-to-day open decision-making and deliberation, empowerment, equality, communication and openness. The EAB can provide advice on this, but to ensure ethical governance, ethical reflection must go beyond this, into the ethical deliberation of everyday practices. Including ethics at different stages of the app development process speaks to horizontal layers of intervention, which empowers individuals, bringing them together to have open deliberations within the context of specific ethical issues, thereby promoting the evolution of appropriate resolutions (Montgomery and Williams, 2019). The vertical governance processes presented in our findings hindered these approaches and stifled ethical discussion because it did not favour a freer flow of ethical deliberation across the different actors involved. As noted above, ethics is a process not a solution, and the process of developing governance structures is one aspect of ethical governance. Our findings suggest that a commitment to ethics requires a commitment to horizontal governance and the free flow of ethical deliberation across a range of non-hierarchal actors.

Third, at least some of the inadequate accountability our interviewees highlighted was justified on the basis of the pandemic emergency situation and the need to prioritise “saving lives” (Gould and Lewis, 2020). In this consequentialist approach, the perceived imminent threat of the virus (which at the time was uncertain, but had catastrophic potential), was so great that when weighed up against the perceived expected benefit of the app (which was hoped would save lives (Gould and Lewis, 2020)), it gave permission for some processes to be established or ensued that would otherwise be considered sub-optimal. For example, communication with the public and with the EAB was not always clear and often deprioritised compared to the app’s technical development. However, these sub-optimal approaches have been argued against in the public health literature. Here, scholars have been aware of the difficult and complex governance issues that emerge during public health emergencies, including the enormous political pressure that policymakers may be under to make decisions (Asgary, 2015; Fenton *et* *al*., 2015), often at fast pace (McLean, 2012). Similar to a number of our own interviewees, these scholars have called for governance processes that promote ethically optimal processes around accountability, openness, transparency and information exchange to be developed ahead of pandemic situations (WHO, 2006; Meltzer Henry, 2019; Mathur, 2020). In fact, in 2007, to ensure ethically defensible decisions were made during public health emergencies, the UK’s National Committee on Ethical Aspects of Pandemic Influenza (CEAPI) developed an ethical framework for responding to such events (Montgomery and Williams, 2019, Committee on Ethical Aspects of Pandemic Influenza, 2007). In October 2019, before the coronavirus pandemic, this committee was effectively re-established as a Moral and Ethical Advisory Group [2]. However, despite this prior ethical work, these principles were not always operationalised around decision-making with relation to the app’s development and governance. The reason for this, we suggest, is that being ethically prepared requires more than just being aware of ethical issues and developing frameworks, but also requires the *ability* and *willingness* [3] of actors who are developing and governing the technology to enact their insights; to be properly ethically prepared, actors involved and making decisions about innovation/implementation processes need to have the *capability, opportunity* and *motivation* to change their behaviour in line with this ethical awareness [4]. Looking forward, the integration of ethics into organisational practice should focus on exploring how to create the conditions to ensure such behaviour change.

In conclusion, in line with the literature that has explored organisational commitments to ethics, our findings highlighted that the EAB achieved some success in integrating ethics into the app development process, particularly at the micro level, but this was restricted by various factors, including a lack of accountability, horizontal governance and the motivation, opportunity and capability of the organisation to change their behaviour in line with ethical awareness. The analysis of this case study suggests that to ensure promises of embedding ethics in future initiatives, more macro level integration of ethics is required into development and decision-making processes. Though we caution that this integration would not have been solutionist; we must be realistic about what we can and should ask ethics to do.

## Supplementary Material

Appendix

## Notes

1 For example, the organisational structure of Genomics England and UKBiobank. Here, the Chairs of the ethics committees are also Board members.

2 https://www.gov.uk/government/groups/moral-and-ethical-advisory-group

3 The authors are grateful to xxx for bringing these concepts/aspects to their attention.

4 This relates to the COM-B model in psychology. The relationship between COM-B and ethical preparedness will be the feature of a forthcoming paper one of the authors is currently working on.
