# Supplementary material for: Bringing ethics into governance: the case of the UK COVID-19 contact tracing app
Source: Int J Health Gov. Author manuscript; Available in PMC 2024 Oct 16. (PMC7616708; doi:10.1108/ijhg-04-2021-0042)
Supplement: Appendix [file EMS199146-supplement-Appendix.pdf]

Appendix
Interview schedule

For non-EAB members

General

(1) Can you tell me your background and how this led to your involvement in the NHSX contact tracing app?

(2) Can you talk me through what your exact involvement in the app has entailed on a day-to-day basis?

Personal/professional views about the app

(1) What do you see as the role of this app in the response to the pandemic?

Developing and implementing the app

(1) Can you describe what technical or other issues, if any, you've come across (or know others have come across) during the development and/or implementation of the app?

(2) If you are aware of this, how have these issues been addressed?

(3) In your opinion, how have these challenges compared to those in other research or work that you/others do?

Making ethics decisions about the app

(1) What broad ethical and/or social concerns or worries do you have about the app?

(2) What reflections do you have on how the ethical issues related to the app compare to those related to contact tracing (non app related) more broadly?

(3) What types of discussions have you been involved in, if any, that have talked about the ethical issues associated with the app?

(4) What resources are you aware of – for example, guidelines, individuals, committees or organisations–that were drawn upon to support ethical decision-making with regard to the app?

(5) In your professional opinion, in these discussions, what decisions were made about the ethical issues and who made them?

(6) Are there any aspects about the app that you feel felt, or felt, uneasy about from an ethical point of view? Why, why not?

(7) In your opinion, do you feel the ethical governance of the app was adequate? Why, why not?

(8) How would you have improved the ethical governance?

(9) Could you describe the governance of the app (who answered to who, who made the decisions, did this change over time) and your reflections on it?

(10) What are your reflections on how the governance of the app compares to that of the test and trace initiative?

(11) In your opinion, how do you think the whole issue of the app has been dealt with by the government? What could the gov have done better?

(12) Moving forward what are the best ways of addressing the concerns we have discussed in this interview?

For EAB members

General

(1) Can you tell me your background and how this led to your involvement in the EAB for the NHSX contact tracing app?

(2))Can you talk me through what your exact involvement in the EAB is, and what this has entailed on a day-to-day basis?

Regarding the EAB

(1) Could you describe to me how and when the EAB was set up?

(2) Could you describe the remit of the EAB, how many times do you meet and who decides what to discuss?

(3) What types of discussions have the EAB had?

(4) What discussions have the EAB had, if any, regarding an exit strategy for the app?

(5) Regarding the discussions the EAB have, how do the concerns about the app fit into the general test/trace strategy? What is exceptional about the app in this process? Why does it add an extra level of ethical concern? Should it?

(6) Where does the EAB get their information from about the app to support your discussions, and what information is this?

(7) What resources are you aware of – for example, guidelines, individuals, committees or organisations–that were drawn upon to support ethical decision-making?

(8) At the end of the EAB meetings, who writes the report and what happens to it?

(9) Does the EAB have decision-making power, and if so, what?

(10) Beyond the EAB, in your professional opinion who has made the final decisions about the app?

(11) Finishing up talking about the EAB, what do you feel has worked and what has not worked in the EAB?

Personal/professional views about the app

(1) What do you see as the role of this app in the response to the pandemic?

(2) What concerns or worries do you have about the app?

(3) In your opinion, do you feel the oversight mechanism for the development of the app was adequate? Why, why not?

(4) How would you have improved the oversight mechanism?

(5) Do you know anything about the oversight mechanism that was put in place for the implementation of the app (eg evaluation beyond the technical issues)? Could you describe these to me?

(6) In your opinion, and only if you feel comfortable talking about it, how do you think the whole issue of the app has been dealt with by the government? What could the government have done better?

(7) Moving forward what are the best ways of addressing the concerns we have discussed in this interview?

For instructions on how to order reprints of this article, please visit our website:
www.emeraldgrouppublishing.com/licensing/reprints.htm
Or contact us for further details: **permissions@emeraldinsight.com**
